# Interventional clinical trials registered per million population per country worldwide

**DOI:** 10.1186/s13063-025-09185-y

**Published:** 2025-11-25

**Authors:** Jonas Leth Bjerg, Dimitrinka Nikolova, Christian Gluud

**Affiliations:** 1 Copenhagen Trial Unit, Centre for Clinical Intervention Research, The Capital Region, Copenhagen University Hospital — Rigshospitalet, Copenhagen, Denmark; 2 Cochrane Hepato-Biliary Group, Copenhagen Trial Unit, Centre for Clinical Intervention Research, The Capital Region, Copenhagen University Hospital — Rigshospitalet, Copenhagen, Denmark; 3 Department of Regional Health Research, The Faculty of Health Sciences, University of Southern Denmark, Odense, Denmark

## Abstract

**Background:**

Sweden and Denmark are amongst the countries which led the world in clinical trial publications per million population. We need, however, to also assess clinical research activities through trial registration rather than publication alone. Enforceable policies for trial registration before launch facilitated global and national clinical research assessments. The World Health Organisation (WHO) hosts the International Clinical Trials Registry Platform (ICTRP) for interventional clinical trials, with data from the year 1999 and onwards.

**Aims:**

To identify the number of interventional clinical trials registered per million population per country worldwide during the 2009- to 2022-year period, and to examine whether registration activity increased in those years when international registration requirements became more widely adopted as compared to the 1999- to 2022-year period.

**Methods:**

We used the WHO ICTRP to extract the yearly numbers of registered interventional clinical trials. We entered the data in Microsoft Excel. We analysed the two periods of years: from 2009 to 2022 with more complete registration of trials and from the whole period 1999 to 2022 with a slow catch-up. To obtain the trial registration activity by country, we divided the total number of interventional clinical trials registered by the country’s population in millions. Information on population numbers originated from the World Bank, the Central Intelligence Agency World Factbook, and The National Institute of Statistics and Economic Studies (of France) as of 2022. We collected data as of 03-01-2024.

**Results:**

In February 2022, there were 943,113 interventional clinical trials registered by country on the ICTRP. After excluding 32,309 trials with unknown country of origin, we analysed 910,804 trials registered in 229 countries and territories. For countries or territories with populations over 1 million, the top five in interventional clinical trial registrations per million population during the 2009- to 2022-year period were Denmark, Estonia, Belgium, The Netherlands, and New Zealand. For the 1999- to 2022-year period, these were Denmark, Estonia, Belgium, The Netherlands, and Latvia. The top five countries or territories, irrespective of population, for both periods were Pitcairn, The Vatican, Tokelau, Niue, and Denmark.

**Conclusions:**

Since 1999, there has been worldwide growth in interventional clinical trial registrations, especially amongst top-contributing countries. The global average stands at approximately 97 registered trials per million people from 2009 to 2022, highlighting the need for continued efforts to improve trial registration and production. Smaller Western countries lead in interventional clinical trials per million population.

**Supplementary Information:**

The online version contains supplementary material available at 10.1186/s13063-025-09185-y.

## Background

Assessment of countries’ contribution to global clinical research was conducted previously by identifying the number of articles published on clinical trials [1, 2]. From 1945 until 2005, the USA and the UK were forerunners in the total number of publications on randomised clinical trials ignoring inhabitant numbers [1] while Sweden and Denmark were leading with 891 and 864 publications on randomised clinical trials per million population during that same period of time [1]. In 2011, Wolff and colleagues revisited the topic on the number of publications on randomised clinical trials, and results matched the previously observed numbers [2]. All results are of interest, but due to the observation that about every second randomised clinical trial is never published [3], national research activities may not become properly gauged. Hence, we found it important to also identify the number of interventional clinical trials registered per country and per million population since 1999.

The negative impact of publication bias has led to activities to reduce clinical research waste [4–7]. The Food and Drug Administration Modernization Act section 113 (FDAMA 113) was introduced in the USA in 1997 [8] demanding protocol registration before the recruitment of participants in trials concerning serious or life-threatening diseases [9]. It was one of the early steps towards proper trial registration. In 2004, the International Committee of Medical Journal Editors (ICMJE) demanded trial registration at or before the onset of enrolment of participants by July 2005 [10]. In 2005, at the Ministerial Summit on Health Research in Mexico, the World Health Organisation (WHO) called upon the global scientific community, international partners, the private sector, civil society, and other relevant stakeholders to ‘*establish a platform linking clinical trials registers in order to ensure a single point of access and the unambiguous identification of trials*’ [11]. These actions, together with activities like the AllTrials initiative, The Cochrane Collaboration, and many others, have been of great importance to oppose publication bias and promote an increase in registrations of unfinished or abandoned trials due to mostly unsatisfactory results [12–16].

The ethical obligation to register and publish clinical trials has also been reinforced by the World Medical Association’s Declaration of Helsinki, lastly revised in 2020. The revision emphasises that studies involving human participants must be registered before recruitment and that trial results must be made publicly available, regardless of outcome [17]. These principles are directly aligned with the goals of transparency and accountability that underpin our present study. Herein, we assess all registered interventional clinical trials in the WHO International Clinical Trial Registry Platform (ICTRP), enabling the possibility to bring forward the individual country’s interventional clinical trial registration per million population [18].

## Aims

To identify the number of interventional clinical trials registered per million population per country worldwide during the 2009- to 2022-year period, and to examine whether registration activity increased in those years when international registration requirements became more widely adopted as compared to the 1999- to 2022-year period.

## Methods

### Data sources and data extraction

The ICTRP, established in 2005, gathers worldwide data on clinical trial registrations from the WHO primary and partner registries (20 in total), data providers, and registries working with the ICTRP towards becoming primary registries (https://www.who.int/tools/clinical-trials-registry-platform/network/data-providers). The ICTRP database contains numbers on the yearly registered interventional clinical trials for the period 1999 to 2022 [18]. We extracted the number of interventional clinical trial registrations by country by selecting the ‘interventional clinical trials’ term from a list of options, followed by searches for (1) trial registrations during the years 1999 to 2022 (the whole available period) and (2) only for the years 2009 to 2022. The year 2009 was provisionally selected, allowing the countries time to adapt to the ICMJE request for registration of trials from 2005 [10]. We acknowledge that trial registration before the year 2009 was less consistent across countries. Therefore, we regarded the data from the 2009- to 2022-year period as more robust and valid, and retained the 1999- to 2022-year period for historical comparison. We selected the two time periods by marking the wanted period in chart A on the Global Observatory on Health Research and Development website, obtaining the numbers on countries from chart E [18]. We copied the numbers from the database into an Excel worksheet, enabling the possibility to obtain a visual representation when comparing the numbers of registered interventional clinical trials with populations in different countries. We used Excel to divide the number of interventional clinical trials by the respective country’s population in millions to obtain the number of registered trials per million population. Therefore, the extracted trial data are obtained solely from the WHO ICTRP. According to the WHO ICTRP, the country variable is assigned based on the recruitment country or countries as self-identified in the primary registries. If a trial recruits in several countries, it is counted once for each recruitment country. Therefore, the present analysis reflects recruitment country rather than sponsor country or registry location.

We gathered data on population sizes for each country, primarily from the World Bank [19] and the Central Intelligence Agency (CIA) World Factbook [20]. We found data for the French colonies at The National Institute of Statistics and Economic Studies (of France) (French Guiana, Guadeloupe, Martinique, Mayotte, Réunion) [21].

### Data analysis

We planned two rankings based on country population. The first included only countries with more than 1 million population. The second included all countries and territories. It must be noted that due to the smallness of some countries and territories, their metric of the number of registered interventional clinical trials per year per million population is multiplied by 1 million, and therefore, the numbers may be less precise.

We handled all numbers and graphs in Microsoft Excel.

## Results

### Description of search and data selection

There were 943,113 registered interventional clinical trials on the WHO ICTRP in February 2022 (the time of last data entry). After excluding 32,309 trials with unknown country of origin, we were left with a total of 910,804 trials, with trial registrations belonging to 229 countries and territories. JB entered the data for analysis in Excel, in conformity with our methods section. DN performed a random data check to validate the correctness of data entry. JB calculated the registration activity of each country by dividing the amount of interventional clinical trials registered by the country’s population and multiplying by 1,000,000.

Dividing the total number of interventional clinical trial registrations (*n* = 910,804) by the world population in millions (*n* = 7.9 billion in 2022) gave a global mean of 96.7 trials per million for the 2009- to 2022-year period (*n* = 766,115) and 115 trials per million for the 1999 to 2022 period.

### Top countries with populations of 1 million population or above

**Fig. 1:**
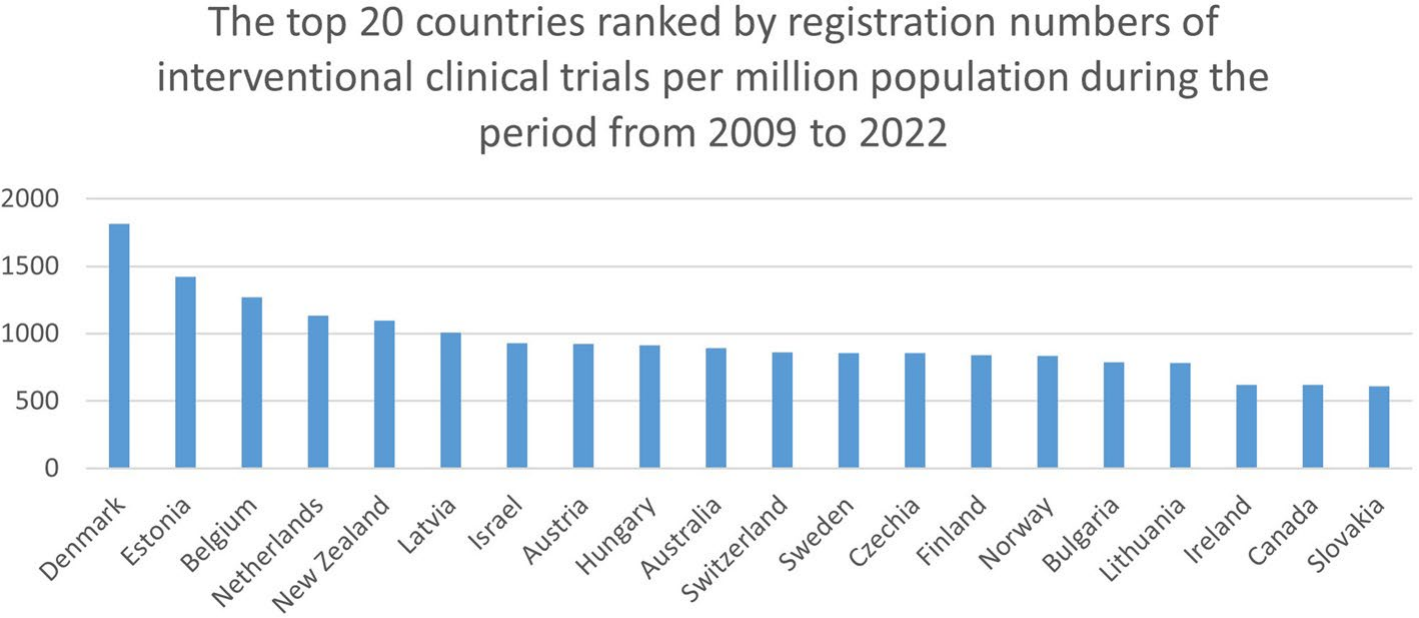
The top 20 countries ranked by registration numbers of interventional clinical trials per million population during the period from 2009 to 2022

**Fig. 2:**
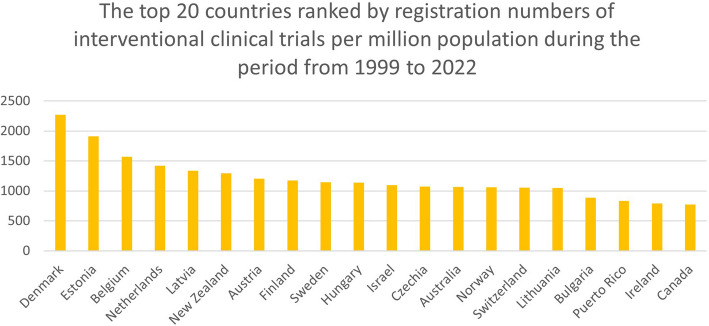
The top 20 countries ranked by registration numbers of interventional clinical trials per million population during the period from 1999 to 2022

Figures 1 and 2 show the top 20 countries with the highest number of registrations of interventional clinical trials amongst countries with a population of 1 million and above for the two year-periods. During the 2009- to 2022-year period, Denmark, Estonia, Belgium, The Netherlands, and New Zealand led the ranking of countries with the highest number of trials registrations. During the 1999- to 2022-year period, Denmark, Estonia, Belgium, The Netherlands, and Latvia were the forerunners.

The number of registered interventional clinical trials in Denmark during the 2009- to 2022-year period was 1815.7 (average 139.7 per year) per million population, and during the 1999- to 2022-year period it was 2267.8 per million population (average 98.6 per year). For Estonia, the number of registered interventional clinical trials during the 2009- to 2022-year period was 1420.5 per million population (average 109.3 per year), and during the 1999- to 2022-year period it was 1910.5 per million population (average 83.1 per year). For Belgium, the number of registered interventional clinical trials during the 2009- to 2022-year period was 1270.6 per million population (average 97.8 per year), and during the 1999- to 2022-year period it was 1568.4 per million population (average 68.2 per year).

Supplementary Table 1 shows the number of interventional clinical trials per million population in the top 20 countries with a population of 1 million or more.

### Top countries amongst all countries and territories

**Fig. 3:**
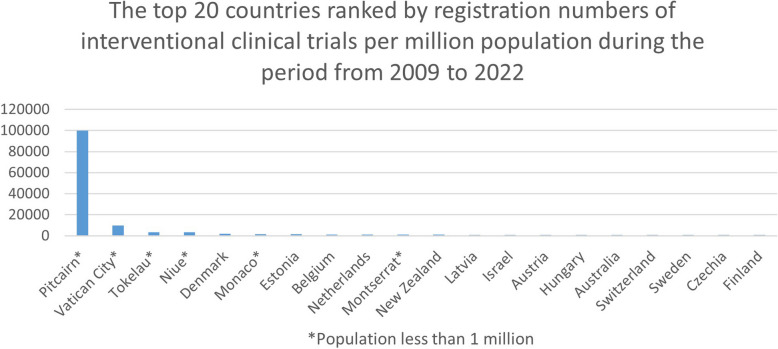
The top 20 countries ranked by registration numbers of interventional clinical trials per million population during the period from 2009 to 2022. Including countries with a population of less than 1 million

**Fig. 4:**
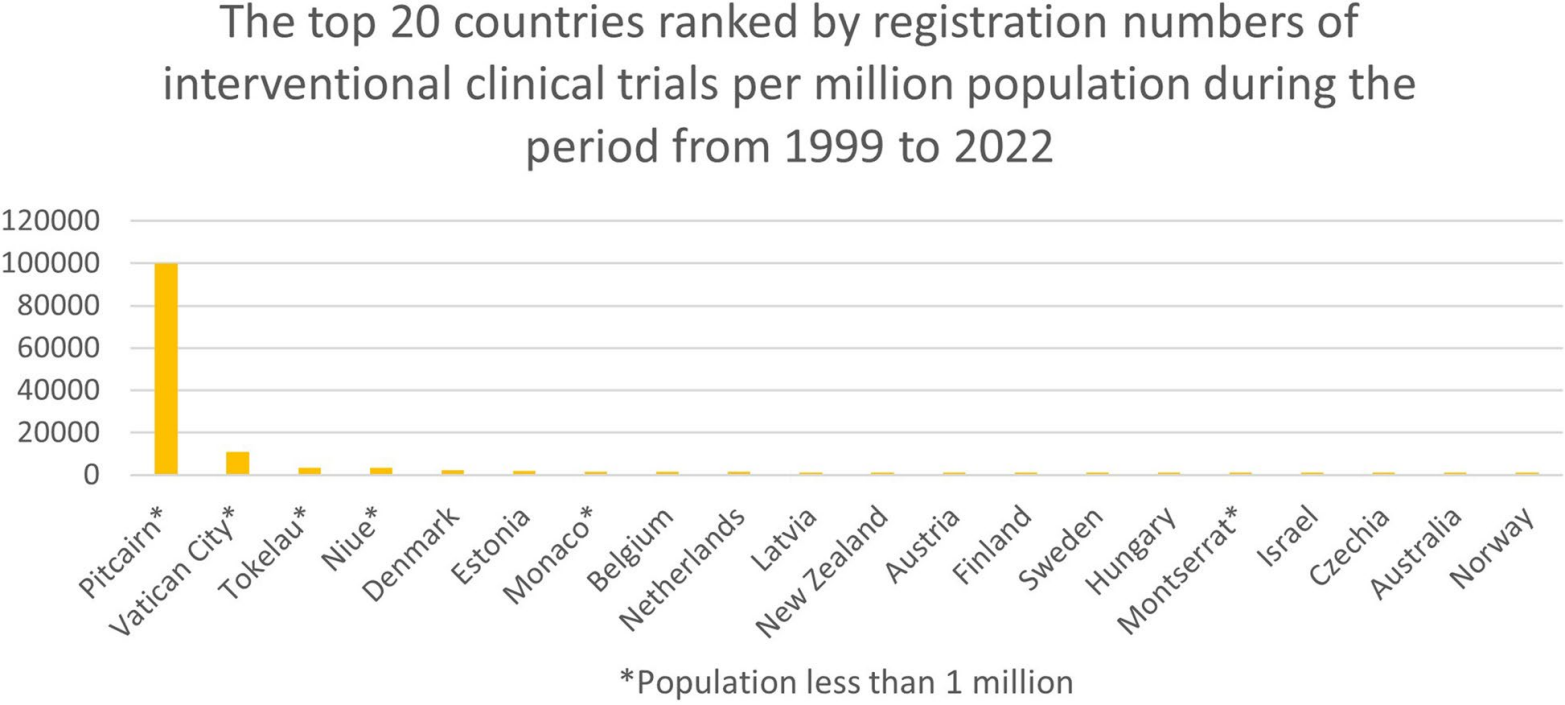
The top 20 countries ranked by registration numbers of interventional clinical trials per million population during the period from 1999 to 2022. Including countries with a population of less than 1 million

We also produced an overview of the top 20 countries and territories regarding interventional clinical trial registrations during the same two periods, 2009 to 2022 and 1999 to 2022 (see Figs. 3 and 4). Pitcairn (territory of the UK), The Vatican, Tokelau (territory of New Zealand), Niue (self-governing in free association with New Zealand), and Denmark showed to be the forerunners in both time periods.

Supplementary Table 2 displays the number of registered interventional clinical trials per million population in the top 20 countries and territories, irrespective of population size.

For the 2009- to 2022-year period, we obtained the following results on the number of registered interventional clinical trials: for Pitcairn, it was 100.000 trials per million population (average 7692.3 per year) based on 5 trials and 50 people; for the Vatican, it was 10,000 trials per million population (average 769.2 per year) based on 10 trials and 1000 people; and for Tokelau, it was 3643 trials per million population (average 280.2 per year) based on 6 trials and 1647 people.

For the 1999- to 2022-year period, we obtained the following results on the number of registered interventional clinical trials: for Pitcairn, no change; for the Vatican, 11,000 trials per million population (average 478.3 per year) based on 11 trials and 1000 people; and for Tokelau, no change.

Data on all countries, present in the ICTRP, is available in Supplementary Table 3.

## Discussion

### Summary of findings

When not accounting for small population countries or territories (≤1 million), Denmark came out as the country with the highest number of trial registrations, according to our unit of measurement. The comparisons of the two periods showed that the number of registered interventional clinical trials picked up significantly during the 2009- to 2022-year period compared to the entire period. For all countries and territories, Pitcairn was the only top contributor of interventional clinical trials when the number of registered trials was held against its population (50 people in 2021). In both scenarios, with and without countries with smaller populations, the ranking of forefront countries remained unchanged in both year intervals.

### Limitations and strengths

Our study aimed to give an account of the interventional clinical trials registration progress by incorporating numbers on all countries, without analysing each trial’s scope, design, and utility. This approach results in a limitation where results do *not* rely on factors, such as sample size and trial quality. To consider the scale (the sample size of a trial) and their methodological quality is important because a high number of trial registrations per million population may result in increased production of smaller-scale trials [2]. An estimate of only the number of registered trials per million population could therefore favour smaller countries conducting many small-scale trials. Furthermore, not all registered trials are initiated or completed. Some records may stand for trials that remain in registries without being initiated or finalised. The extent of this problem is likely to differ between countries, which is highly likely to affect comparability.

Second, limitations in this study do also repeat the limitations of the ICTRP. Some registered trials had missing data, resulting in a relatively larger number of trials with an unknown country of origin (*n* = 32,309). As this corresponded to less than 5% of the interventional clinical trials, we considered the missing data spread evenly across all countries, not affecting the overall result. While dividing the number of registered interventional clinical trials by a country’s population in millions, we encountered a misrepresentation when a population was too small. Therefore, we used a lower limit of 1 million population, highlighting results of 69 countries (6 of which are present in Figs. 3 and 4).

Third, the counts for some countries could have been inflated because of the possibility of cross-registration of the same trial in multiple registries or multiple country sites. The ICTRP addresses this by deduplicating records using parent/child trial flags and synchronising investigator reported information, as described in their guidance on unambiguous trial identification [22]. While some duplication cannot be excluded, we assume the impact on our overall findings would be minimal.

Fourth, our data on country populations are a snapshot of a certain point in time and are not representative for the entire 1999- to 2022-year period. Though the world population is growing [23], some countries, especially in eastern Europe, are experiencing population decline [24]. Therefore, one should be aware that some of the mentioned forefront countries could credit their number of registered interventional trials per million population to a population decrease as this results in an increased position. Similarly, countries that have had an increase in population obtain a lower number of registered interventional trials per million population.

Our study did not assess trial protocol changes or modifications made within trials registries after initial registration. Such amendments may affect research integrity and the interpretation of outcomes. Analysing the frequency and impact of these changes would be a valuable direction for future research.

We followed our aims and present results based on the net registration of interventional clinical trials. Sufficiently powered trials are with a higher value than insufficiently powered trials, and one may ponder whether countries with large populations, like China or the USA, could have registered fewer but larger trials compared to small top countries such as Denmark. This is worth investigating further.

As mentioned, our study aimed to include all available data on trial registration from every country in the world, also counting colonies and territories with independent data (*n* = 229). It is hard to determine whether we have succeeded in this, but we recognise the strength in using the data made available by the WHO through their ICTRP.

### Results in context

The results presented in this paper show that there have been countries consistently in the forefront of trial registration and hopefully production, but also that previously unacknowledged countries have risen, becoming some of the top contributors. It should be acknowledged that the observed increase over time may reflect improved registration practices rather than a true proportional increase in trial production. Nonetheless, we consider this to be an important part of our findings, as it underlines both progress in registration practices and the continuous need for stronger enforcement of prospective trial registration.

Countries like Estonia, Czechia, Lithuania, Bulgaria, Puerto Rico, and Ireland have gone from not even making the top 20 list on trial publications in the year-period 1946–2005 [1], to setting an example that others should aim for today. Especially, Estonia has made a big progress in the last 23 years. While formerly being at the very top of contributors, Sweden has fallen a few places in the top 20 ranking countries compared with the results of two articles on publication numbers of randomised and controlled trials [1, 2]. Though not to neglect that Sweden, amongst others, is still a big contributor on the international scene of interventional clinical trials.

Though Denmark has been recognised as the best performing country in our article in terms of clinical trial registrations, there is room for improvement. Based on a report published in TranspariMED in February 2024 [25], the Scandinavian countries had a lack of reporting trial results. Denmark, at the time of the report, still had 19% unpublished trial results for trials registered in the year period 2016 to 2019.

Even though countries had high inputs in registrations and productions of interventional clinical trials during our overall studied period, we recognise that the numbers could be higher. Countries should naturally aim for a 100% publication of trial results, as this activity most importantly also follows ethical principles for medical research involving humans (https://www.wma.net/policies-post/wma-declaration-of-helsinki-ethical-principles-for-medical-research-involving-human-subjects/).

A substantial proportion of registered interventional clinical trials remain unpublished, raising concerns about research transparency and publication bias. Meta-analyses conducted prior to 2020 estimated that approximately 54% of registered trials were published within 24 months of completion [26]. A Cochrane review from 2024 found that only about 50% of over 160,000 registered trials had published their results, highlighting a significant and continuing transparency gap [27]. Further recent studies indicated that these figures did not significantly improve. For instance, a study on glaucoma found that only 53% of the trials were published, and only 23% of these were published within the 12-month mandated reporting period [28]. Similarly, a study on motor disorders reported publication rates ranging from 39 to 67% [29]. These findings underline the persistent gap between trial registration and publication, highlighting the need for enhanced enforcement of reporting requirements to ensure research findings are disseminated and contribute to evidence-based healthcare.

Highly populated areas are India with 1,417,173,170 population, China with 1,412,175,000 population, and the continent of Africa with 1,332,006,610 population. These populations together correspond to about 50% of our global population. Looking at the interventional clinical trial registrations per million population during the 2009- to 2022-year period (China = 43; India = 27; and Africa = 15), the disproportion in the distribution of trial registrations is obvious considering the world mean of 97 registered interventional clinical trials per million population during the same period of time.

## Conclusions

Looking at our two timeframes of measurement, we expected to see a big increase in registered trials produced in the year-period of 2009 to 2022, and so we did. The three forefront countries Denmark, Estonia, and Belgium all had a big increase in trial production in the mentioned period, with Denmark registering 10,718 interventional clinical trials in the 2009- to 2022-year period from a total of 13,387 in the 1999- to 2022-year period. The same tendency was seen with Estonia (*n* = 1916 in 2009 to 2022, *n* = 2577 in 1999 to 2022) and Belgium (*n* = 14,848 in 2009 to 2022, *n* = 18,328 in 1999 to 2022). This increase in registration of trials could be a result of the common advancement and development in health research, but we may also conclude that this increase proceeds from the ICMJE’s requirement of trial registration along with other initiatives. Though unregistered trials are still present [30], we are nevertheless seeing an increase in registrations. Large countries, and the largest world economies, like the USA, China, and Japan [31], had the overall highest production of interventional clinical trials in the 2009- to 2022-year period, but these numbers tend to fade into smaller proportions after undergoing our method of analysis (USA = 329 registered trials per million population, China = 43 registered trials per million population, Japan = 349 registered trials per million population). This emphasises the importance of a standardised approach and incentives for trial data sharing.

Since 1999, the number of interventional clinical trial registrations has been increasing globally, and for many countries which are amongst the top contributors, the registrations have increased almost exponentially. During the 23 -year period that our study examines, several countries have managed to register thousands of interventional clinical trials. Whatever the reason is for the increase in registrations, e.g. ICMJE recommendations, it is an increase of major importance for obtaining more complete data on healthcare interventions [32]. In 2007, we suggested a benchmark of 700 trials per million population per country in 60 years (equal to about 12 trials per million per year), which would have resulted in millions of trials [1]. Currently, 15 years later, the number of registered interventional clinical trials has become 115 per million population worldwide per 23 years (i.e. the total number of registered trials (*n* = 910,804) divided by the world population in millions in the 1999- to 2022-year period). This is equal to about five trials per million population per year. This shows that even though we are seeing an increase in trial registration by country, we must keep pursuing methods for a more thorough trial registration and trial production.

## Supplementary Information

Supplementary Material 1.

Supplementary Material 2.

Supplementary Material 3.

## Data Availability

All data generated or analysed during this study are included in this published article [and its supplementary information files].
