# Supplementary material for: Interventional clinical trials registered per million population per country worldwide
Source: Trials. 2025 Nov 25;26:543. doi: 10.1186/s13063-025-09185-y (PMC12648775; doi:10.1186/s13063-025-09185-y)
Supplement: Supplementary file 1 — Supplementary Material 1. [file 13063_2025_9185_MOESM1_ESM.docx]

| **Country** | **Population** | **Registered interventional clinical trials 1999-2022** | **Registered interventional clinical trials 2009-2022** | **Registered interventional clinical trials pr. mil. population 1999-2022** | **Registered interventional clinical trials pr. mil. population 2009-2022** |
| --- | --- | --- | --- | --- | --- |
| Denmark | 5,903,040 | 13,387 | 10,718 | 2,267.8 | 1,815.7 |
| Estonia | 1,348,840 | 2,577 | 1,916 | 1,910.5 | 1,420.5 |
| Belgium | 11,685,810 | 18,328 | 14,848 | 1,568.4 | 1,270.6 |
| Netherlands | 17,700,980 | 25,177 | 20,088 | 1,422.4 | 1,134.9 |
| Latvia | 1,879,380 | 2,515 | 1,899 | 1,338.2 | 1,010.4 |
| New Zealand | 5,124,100 | 6,637 | 5,615 | 1,295.3 | 1,095.8 |
| Austria | 9,041,850 | 10,872 | 8,358 | 1,202.4 | 924.4 |
| Finland | 5,556,110 | 6,514 | 4,670 | 1,172.4 | 840.5 |
| Sweden | 10,486,941 | 12,013 | 8,981 | 1,145.5 | 856.4 |
| Hungary | 9,643,050 | 10,981 | 8,808 | 1,138.7 | 913.4 |
| Israel | 9,557,500 | 10,464 | 8,880 | 1,094.8 | 929.1 |
| Czechia | 10,672,120 | 11,439 | 9,125 | 1,071.9 | 855.0 |
| Australia | 26,005,540 | 27,670 | 23,205 | 1,064.0 | 892.3 |
| Norway | 5,457,130 | 5,783 | 4,543 | 1,059.7 | 832.5 |
| Switzerland | 8,775,760 | 9,250 | 7,580 | 1,054.0 | 863.7 |
| Lithuania | 2,831,640 | 2,964 | 2,219 | 1,046.7 | 783.6 |
| Bulgaria | 6,465,100 | 5,742 | 5,093 | 888.2 | 787.8 |
| Puerto Rico | 3,221,790 | 2,691 | 1,778 | 835.2 | 551.9 |
| Ireland | 5,127,170 | 4,058 | 3,191 | 791.5 | 622.4 |
| Canada | 38,929,900 | 30,168 | 24,118 | 774.9 | 619.5 |

Table 1. Registered interventional clinical trials amongst top 20 countries with a population of 1 million or above. *Population less than 1 million
