# Supplementary material for: Interventional clinical trials registered per million population per country worldwide
Source: Trials. 2025 Nov 25;26:543. doi: 10.1186/s13063-025-09185-y (PMC12648775; doi:10.1186/s13063-025-09185-y)
Supplement: Supplementary file 3 — Supplementary Material 3. [file 13063_2025_9185_MOESM3_ESM.docx]

| **Country** | **Population** | **Registered interventional clinical trials 1999-2022** | **Registered interventional clinical trials 2009-2022** | **Registered interventional clinical trials per mil. population 1999-2022** | **Registered interventional clinical trials per mil. Population 2009-2022** |
| --- | --- | --- | --- | --- | --- |
| Pitcairn* | 50 | 5 | 5 | 100,000.0 | 100,000.0 |
| Vatican City* | 1,000 | 11 | 10 | 11,000.0 | 10,000.0 |
| Tokelau* | 1,647 | 6 | 6 | 3,643.0 | 3,643.0 |
| Niue* | 2,000 | 7 | 7 | 3,500.0 | 3,500.0 |
| Denmark | 5,903,040 | 13,387 | 10,718 | 2,267.8 | 1,815.7 |
| Estonia | 1,348,840 | 2,577 | 1,916 | 1,910.5 | 1,420.5 |
| Monaco* | 36,470 | 64 | 57 | 1,754.9 | 1,562.9 |
| Belgium | 11,685,810 | 18,328 | 14,848 | 1,568.4 | 1,270.6 |
| Netherlands | 17,700,980 | 25,177 | 20,088 | 1,422.4 | 1,134.9 |
| Latvia | 1,879,380 | 2,515 | 1,899 | 1,338.2 | 1,010.4 |
| New Zealand | 5,124,100 | 6,637 | 5,615 | 1,295.3 | 1,095.8 |
| Austria | 9,041,850 | 10,872 | 8,358 | 1,202.4 | 924.4 |
| Finland | 5,556,110 | 6,514 | 4,670 | 1,172.4 | 840.5 |
| Sweden | 10,486,941 | 12,013 | 8,981 | 1,145.5 | 856.4 |
| Hungary | 9,643,050 | 10,981 | 8,808 | 1,138.7 | 913.4 |
| Montserrat* | 5,414 | 6 | 6 | 1,108.2 | 1,108.2 |
| Israel | 9,557,500 | 10,464 | 8,880 | 1,094.8 | 929.1 |
| Czechia | 10,672,120 | 11,439 | 9,125 | 1,071.9 | 855.0 |
| Australia | 26,005,540 | 27,670 | 23,205 | 1,064.0 | 892.3 |
| Norway | 5,457,130 | 5,783 | 4,543 | 1,059.7 | 832.5 |
| Switzerland | 8,775,760 | 9,250 | 7,580 | 1,054.0 | 863.7 |
| Lithuania | 2,831,640 | 2,964 | 2,219 | 1,046.7 | 783.6 |
| Iceland* | 382,000 | 340 | 244 | 890.1 | 638.7 |
| Bulgaria | 6,465,100 | 5,742 | 5,093 | 888.2 | 787.8 |
| Puerto Rico | 3,221,790 | 2,691 | 1,778 | 835.2 | 551.9 |
| Tuvalu* | 11,310 | 9 | 9 | 795.8 | 795.8 |
| Ireland | 5,127,170 | 4,058 | 3,191 | 791.5 | 622.4 |
| Canada | 38,929,900 | 30,168 | 24,118 | 774.9 | 619.5 |
| Slovakia | 5,431,750 | 4,049 | 3,305 | 745.4 | 608.5 |
| Cook Islands* | 8,128 | 6 | 6 | 738.2 | 738.2 |
| Palau* | 18,050 | 13 | 12 | 720.2 | 664.8 |
| Singapore | 5,637,020 | 4,014 | 3,414 | 712.1 | 605.6 |
| Saint Barthélemy* | 7,103 | 5 | 5 | 703.9 | 703.9 |
| Slovenia | 2,111,990 | 1,334 | 1,061 | 631.6 | 502.4 |
| Nauru* | 12,670 | 8 | 7 | 631.4 | 552.5 |
| Saint Helena* | 7,925 | 5 | 5 | 630.9 | 630.9 |
| United Kingdom | 66,971,400 | 41,008 | 30,790 | 612.3 | 459.7 |
| Spain | 47,778,340 | 29,209 | 24,253 | 611.3 | 507.6 |
| Greece | 10,426,920 | 6,152 | 5,008 | 590.0 | 480.3 |
| American Samoa* | 44,270 | 26 | 24 | 587.3 | 542.1 |
| Croatia | 3,855,600 | 2,218 | 1,885 | 575.3 | 488.9 |
| France | 67,971,310 | 33,709 | 28,098 | 495.9 | 413.4 |
| Germany | 83,797,990 | 41,077 | 32,375 | 490.2 | 386.3 |
| Portugal | 10,409,700 | 5,056 | 4,024 | 485.7 | 386.6 |
| Italy | 58,940,430 | 26,715 | 20,824 | 453.3 | 353.3 |
| United States | 333,287,560 | 142,300 | 109,567 | 427.0 | 328.7 |
| Serbia | 6,664,450 | 2,747 | 2,473 | 412.2 | 371.1 |
| Poland | 36,821,750 | 15,086 | 13,171 | 409.7 | 357.7 |
| South Korea | 51,628,120 | 20,702 | 19,254 | 401.0 | 372.9 |
| Antigua and Barbuda* | 93,760 | 37 | 37 | 394.6 | 394.6 |
| Iran | 88,550,570 | 34,646 | 33,686 | 391.3 | 380.4 |
| Wallis and Futuna* | 15,891 | 6 | 6 | 377.6 | 377.6 |
| Japan | 125,124,990 | 47,059 | 43,678 | 376.1 | 349.1 |
| Liechtenstein* | 39,330 | 13 | 13 | 330.5 | 330.5 |
| Georgia | 3,712,500 | 1,215 | 1,175 | 327.3 | 316.5 |
| Anguilla* | 18,741 | 6 | 6 | 320.2 | 320.2 |
| San Marino* | 33,660 | 10 | 9 | 297.1 | 267.4 |
| Romania | 19,047,010 | 5,261 | 4,588 | 276.2 | 240.9 |
| Luxemborg* | 653,100 | 170 | 141 | 260.3 | 215.9 |
| Marshall Islands* | 41,570 | 10 | 9 | 240.6 | 216.5 |
| Saint Kitts and Nevis* | 47,660 | 11 | 11 | 230.8 | 230.8 |
| Andorra* | 79,820 | 18 | 16 | 225.5 | 200.5 |
| Gibraltar* | 32,650 | 7 | 7 | 214.4 | 214.4 |
| Chile | 19,603,730 | 3,786 | 3,107 | 193.1 | 158.5 |
| British Virgin Islands* | 31,300 | 6 | 6 | 191.7 | 191.7 |
| Lebanon | 5,489,740 | 1,015 | 938 | 184.9 | 170.9 |
| Republic of Moldova | 2,538,890 | 461 | 448 | 181.6 | 176.5 |
| Guam* | 171,770 | 29 | 29 | 168.8 | 168.8 |
| Cyprus | 1,251,490 | 206 | 189 | 164.6 | 151.0 |
| Saint Martin (French part)* | 31,790 | 5 | 5 | 157.3 | 157.3 |
| Bosnia and Herzegovina | 3,233,530 | 495 | 456 | 153.1 | 141.0 |
| Dominica * | 72,740 | 11 | 10 | 151.2 | 137.5 |
| Faroe Islands* | 53,090 | 8 | 8 | 150.7 | 150.7 |
| Cayman Islands* | 68,710 | 10 | 10 | 145.5 | 145.5 |
| North Macedonia | 2,057,680 | 298 | 260 | 144.8 | 126.4 |
| Argentina | 46,234,830 | 6,570 | 5,455 | 142.1 | 118.0 |
| Greenland* | 56,660 | 8 | 8 | 141.2 | 141.2 |
| Martinique* | 352,205 | 49 | 49 | 139.1 | 139.1 |
| Ukraine | 38,000,000 | 5,108 | 4,573 | 134.4 | 120.3 |
| Turks and Caicos Islands* | 45,700 | 6 | 6 | 131.3 | 131.3 |
| Malta* | 531,110 | 69 | 40 | 129.9 | 75.3 |
| Panama | 4,408,580 | 539 | 413 | 122.3 | 93.7 |
| Northern Mariana Islands* | 49,550 | 6 | 5 | 121.1 | 100.9 |
| Turkey | 84,979,910 | 10,237 | 9,629 | 120.5 | 113.3 |
| Thailand | 71,697,030 | 8,582 | 7,925 | 119.7 | 110.5 |
| Sint Maarten (Dutch part)* | 42,850 | 5 | 5 | 116.7 | 116.7 |
| United States Virgin Islands* | 105,410 | 12 | 12 | 113.8 | 113.8 |
| South Africa | 59,893,890 | 5,779 | 4,640 | 96.5 | 77.5 |
| Isle of man* | 84,520 | 8 | 8 | 94.7 | 94.7 |
| Bermuda* | 63,530 | 6 | 5 | 94.4 | 78.7 |
| Peru | 34,049,590 | 3,195 | 2,274 | 93.8 | 66.8 |
| Guadeloupe* | 378,476 | 35 | 34 | 92.5 | 89.8 |
| Malaysia | 33,938,220 | 3,076 | 2,736 | 90.6 | 80.6 |
| Grenada* | 125,440 | 11 | 10 | 87.7 | 79.7 |
| Costa Rica | 5,180,830 | 452 | 259 | 87.2 | 50.0 |
| Tonga* | 106,860 | 9 | 8 | 84.2 | 74.9 |
| Belize* | 405,270 | 34 | 27 | 83.9 | 66.6 |
| Brazil | 215,313,500 | 17,344 | 15,431 | 80.6 | 71.7 |
| Micronesia* | 114,160 | 9 | 9 | 78.8 | 78.8 |
| Belarus | 9,228,070 | 709 | 659 | 76.8 | 71.4 |
| Egypt | 110,990,100 | 8,355 | 8,103 | 75.3 | 73.0 |
| Bahamas* | 409,980 | 30 | 24 | 73.2 | 58.5 |
| Russian Federation | 144,236,930 | 10,410 | 9,071 | 72.2 | 62.9 |
| Kiribati* | 131,230 | 9 | 9 | 68.6 | 68.6 |
| Saint Vincent and the Grenadines* | 103,950 | 7 | 7 | 67.3 | 67.3 |
| Colombia | 51,874,020 | 3,343 | 2,856 | 64.4 | 55.1 |
| Montenegro* | 617,210 | 38 | 26 | 61.6 | 42.1 |
| Saint Lucia* | 179,860 | 11 | 11 | 61.2 | 61.2 |
| French Guiana* | 296,058 | 18 | 18 | 60.8 | 60.8 |
| Mexico | 127,504,130 | 7,564 | 6,262 | 59.3 | 49.1 |
| Seychelles* | 119,880 | 7 | 6 | 58.4 | 50.1 |
| Gambia | 2,705,990 | 153 | 111 | 56.5 | 41.0 |
| Aruba* | 106,440 | 6 | 6 | 56.4 | 56.4 |
| Qatar | 2,695,120 | 147 | 135 | 54.5 | 50.1 |
| Barbados* | 281,630 | 15 | 15 | 53.3 | 53.3 |
| Botswana | 2,630,300 | 132 | 103 | 50.2 | 39.2 |
| Mauritius | 1,262,520 | 63 | 61 | 49.9 | 48.3 |
| New Caledonia* | 269,220 | 13 | 13 | 48.3 | 48.3 |
| Tunisia | 12,356,120 | 591 | 507 | 47.8 | 41.0 |
| Armenia | 2,780,470 | 127 | 117 | 45.7 | 42.1 |
| China | 1,412,175,000 | 64,194 | 60,968 | 45.5 | 43.2 |
| Guatemala | 17,357,890 | 769 | 604 | 44.3 | 34.8 |
| Réunion* | 869,993 | 38 | 37 | 43.7 | 42.5 |
| Jordan | 11,285,870 | 484 | 453 | 42.9 | 40.1 |
| Samoa* | 222,380 | 9 | 9 | 40.5 | 40.5 |
| Cuba | 11,212,190 | 450 | 327 | 40.1 | 29.2 |
| Brunei Darussalam* | 449,000 | 18 | 18 | 40.1 | 40.1 |
| Vanuatu* | 326,740 | 13 | 12 | 39.8 | 36.7 |
| French Polynesia* | 306,280 | 12 | 11 | 39.2 | 35.9 |
| Bahrain | 1,472,230 | 57 | 53 | 38.7 | 36.0 |
| Gabon | 2,388,990 | 90 | 69 | 37.7 | 28.9 |
| Saudi arabia | 36,408,820 | 1,317 | 1,266 | 36.2 | 34.8 |
| United Arab Emirates | 9,441,130 | 341 | 312 | 36.1 | 33.0 |
| Sao Tome and Principe Africa* | 227,380 | 8 | 8 | 35.2 | 35.2 |
| Eswatini | 1,201,670 | 42 | 40 | 35.0 | 33.3 |
| Curacao* | 150,000 | 5 | 5 | 33.3 | 33.3 |
| Kuwait | 4,268,870 | 141 | 133 | 33.0 | 31.2 |
| Guinea-Bissau | 2,105,570 | 69 | 41 | 32.8 | 19.5 |
| Jamaica | 2,827,380 | 92 | 75 | 32.5 | 26.5 |
| Albania | 2,777,690 | 87 | 81 | 31.3 | 29.2 |
| Oman | 4,576,300 | 140 | 131 | 30.6 | 28.6 |
| Dominican Republic | 11,228,820 | 335 | 273 | 29.8 | 24.3 |
| Uruguay | 3,422,790 | 101 | 60 | 29.5 | 17.5 |
| India | 1,417,173,170 | 40,930 | 39,069 | 28.9 | 27.6 |
| Mayotte* | 299,022 | 8 | 8 | 26.8 | 26.8 |
| Solomon Islands* | 724,270 | 18 | 15 | 24.9 | 20.7 |
| Sri Lanka | 22,181,000 | 532 | 478 | 24.0 | 21.5 |
| Suriname* | 618,040 | 13 | 11 | 21.0 | 17.8 |
| Trinidad and Tobago | 1,531,040 | 32 | 24 | 20.9 | 15.7 |
| Uganda | 47,249,580 | 969 | 836 | 20.5 | 17.7 |
| Fiji* | 929,770 | 19 | 17 | 20.4 | 18.3 |
| Kenya | 54,027,490 | 1,052 | 946 | 19.5 | 17.5 |
| Malawi | 20,405,320 | 392 | 332 | 19.2 | 16.3 |
| Philippines | 115,559,010 | 2,051 | 1,683 | 17.7 | 14.6 |
| El Salvador | 6,336,390 | 110 | 88 | 17.4 | 13.9 |
| Zambia | 20,017,670 | 343 | 281 | 17.1 | 14.0 |
| Cabo Verde* | 593,150 | 10 | 9 | 16.9 | 15.2 |
| Bhutan* | 782,460 | 13 | 13 | 16.6 | 16.6 |
| Kosovo | 1,761,980 | 29 | 29 | 16.5 | 16.5 |
| Guyana* | 808,730 | 13 | 12 | 16.1 | 14.8 |
| Zimbabwe | 16,320,540 | 248 | 213 | 15.2 | 13.1 |
| Lesotho | 2,305,820 | 35 | 35 | 15.2 | 15.2 |
| Ecuador | 18,001,000 | 262 | 164 | 14.6 | 9.1 |
| Paraguay | 6,780,740 | 95 | 87 | 14.0 | 12.8 |
| Mongolia | 3,398,370 | 46 | 38 | 13.5 | 11.2 |
| Maldives* | 523,790 | 7 | 7 | 13.4 | 13.4 |
| Democratic People’s Republic of Korea | 26,069,420 | 326 | 298 | 12.5 | 11.4 |
| Comoros* | 836,770 | 10 | 10 | 12.0 | 12.0 |
| Timor-Leste | 1,341,300 | 16 | 16 | 11.9 | 11.9 |
| Honduras | 10,432,860 | 122 | 99 | 11.7 | 9.5 |
| Ghana | 33,475,870 | 383 | 322 | 11.4 | 9.6 |
| Syrian Arab Republic | 22,125,250 | 252 | 246 | 11.4 | 11.1 |
| Rwanda | 13,776,700 | 156 | 142 | 11.3 | 10.3 |
| Vietnam | 98,186,860 | 1,101 | 1,023 | 11.2 | 10.4 |
| Burkina Faso | 22,889,580 | 247 | 198 | 10.8 | 8.7 |
| Nepal | 30,547,580 | 308 | 280 | 10.1 | 9.2 |
| Pakistan | 235,824,860 | 2,342 | 2,176 | 9.9 | 9.2 |
| Venezuela | 28,301,700 | 281 | 152 | 9.9 | 5.4 |
| Congo | 5,970,420 | 59 | 47 | 9.9 | 7.9 |
| Djibouti | 1,120,850 | 11 | 10 | 9.8 | 8.9 |
| Occupied Palestinian territory | 5,043,610 | 49 | 49 | 9.7 | 9.7 |
| United Republic of Tanzania | 65,497,750 | 579 | 474 | 8.8 | 7.2 |
| Mali | 22,593,590 | 192 | 152 | 8.5 | 6.7 |
| Equatorial Guinea | 1,674,910 | 14 | 14 | 8.4 | 8.4 |
| Lao People’s Democratic Republic | 7,529,480 | 60 | 50 | 8.0 | 6.6 |
| Haiti | 11,585,000 | 90 | 74 | 7.8 | 6.4 |
| Kazakhstan | 19,621,970 | 144 | 138 | 7.3 | 7.0 |
| Sierra Leone | 8,605,720 | 63 | 60 | 7.3 | 7.0 |
| Cambodia | 16,767,840 | 121 | 105 | 7.2 | 6.3 |
| Senegal | 17,316,450 | 123 | 100 | 7.1 | 5.8 |
| Kyrgyzstan | 6,974,900 | 44 | 44 | 6.3 | 6.3 |
| Liberia | 5,302,680 | 32 | 29 | 6.0 | 5.5 |
| Cameroon | 27,914,540 | 168 | 145 | 6.0 | 5.2 |
| Namibia | 2,567,010 | 14 | 13 | 5.5 | 5.1 |
| Morocco | 37,457,970 | 193 | 155 | 5.2 | 4.1 |
| Benin | 13,352,860 | 67 | 56 | 5.0 | 4.2 |
| Bolivia | 12,224,110 | 59 | 42 | 4.8 | 3.4 |
| Papua New Guinea | 10,142,620 | 48 | 43 | 4.7 | 4.2 |
| Mozambique | 32,969,520 | 154 | 127 | 4.7 | 3.9 |
| Iraq | 44,496,120 | 200 | 192 | 4.5 | 4.3 |
| Guinea | 13,859,340 | 56 | 53 | 4.0 | 3.8 |
| Côte d’Ivoire | 28,160,540 | 112 | 101 | 4.0 | 3.6 |
| Nigeria | 218,541,210 | 847 | 810 | 3.9 | 3.7 |
| Bangladesh | 171,186,370 | 639 | 559 | 3.7 | 3.3 |
| Nicaragua | 6,948,390 | 25 | 19 | 3.6 | 2.7 |
| Algeria | 44,903,220 | 160 | 149 | 3.6 | 3.3 |
| Indonesia | 275,501,340 | 979 | 874 | 3.6 | 3.2 |
| Eritrea | 3,684,030 | 12 | 11 | 3.3 | 3.0 |
| Mauritania | 4,736,140 | 14 | 11 | 3.0 | 2.3 |
| Libya | 6,812,340 | 20 | 20 | 2.9 | 2.9 |
| Ethiopia | 123,379,920 | 359 | 340 | 2.9 | 2.8 |
| Central African republic | 5,579,140 | 16 | 15 | 2.9 | 2.7 |
| Azerbaijan | 10,141,760 | 27 | 25 | 2.7 | 2.5 |
| Togo | 8,848,700 | 22 | 20 | 2.5 | 2.3 |
| Sudan | 46,874,200 | 95 | 85 | 2.0 | 1.8 |
| Niger | 26,207,980 | 53 | 48 | 2.0 | 1.8 |
| Burundi | 12,889,580 | 26 | 24 | 2.0 | 1.9 |
| Somalia | 17,597,510 | 35 | 35 | 2.0 | 2.0 |
| Democratic Republic of the Congo | 99,010,210 | 139 | 131 | 1.4 | 1.3 |
| Myanmar | 54,179,310 | 73 | 69 | 1.3 | 1.3 |
| Madagascar | 29,611,710 | 37 | 29 | 1.2 | 1.0 |
| Turkmenistan | 6,430,770 | 8 | 8 | 1.2 | 1.2 |
| Afghanistan | 41,128,770 | 51 | 42 | 1.2 | 1.0 |
| Chad | 17,723,310 | 20 | 19 | 1.1 | 1.1 |
| Tajikistan | 9,952,790 | 11 | 11 | 1.1 | 1.1 |
| Uzbekistan | 35,648,100 | 34 | 30 | 1.0 | 0.8 |
| South Sudan | 10,913,160 | 8 | 8 | 0.7 | 0.7 |
| Angola | 35,588,990 | 26 | 22 | 0.7 | 0.6 |
| Yemen | 33,696,610 | 20 | 18 | 0.6 | 0.5 |

Table 3. All countries from the ICTRP and their respective interventional trial registrations. *Population less than 1 million
